# Research needs related to firearm rights restoration

**DOI:** 10.1186/s40621-023-00482-1

**Published:** 2024-01-05

**Authors:** Julie M. Kafka, Frederick P. Rivara, Rachel Ross, Ali Rowhani-Rahbar

**Affiliations:** 1 Firearm Injury & Policy Research Program, University of Washington, Seattle, WA USA; 2 Department of Pediatrics, Firearm Injury & Policy Research Program, University of Washington, Seattle, WA USA; 3 Department of Epidemiology, Firearm Injury & Policy Research Program, University of Washington, Seattle, WA USA

**Keywords:** Firearms, Violence, Injury, Domestic violence, Policy

## Abstract

**Background:**

In the USA, firearms are commonly involved in many incidents of serious interpersonal harm. Federal law prohibits the purchase and possession of firearms by certain high-risk groups including those with prior felony or domestic violence misdemeanor convictions. Evidence supports the effectiveness of these prohibitions, but little is known about how often prohibited persons later seek to have their firearm rights restored.

**Main body:**

For this commentary, we systematically searched the empirical literature for information about who requests firearm rights restoration in the USA, how often it is granted, and what its consequences are. We found a dearth of empirical literature on this topic.

**Conclusion:**

We call for attention to this gap in the research. There is a need to build an evidence base that can help inform state policy and courtroom practices regarding the eligibility, appropriateness, and risk for subsequent harm following firearm rights restoration among persons who are prohibited based on a criminal conviction history.

**Supplementary Information:**

The online version contains supplementary material available at 10.1186/s40621-023-00482-1.

## Background

Firearms are commonly used in the perpetration of interpersonal harm in the United States (USA). In 2021, firearms were used in 79.2% of homicides, 44.9% of robberies, and 41.5% of aggravated assaults (FBI 2021). When firearms are present during a violent event, odds of fatality more than doubles (Weaver et al. 2004). For individuals with a history of violent behavior, firearm access can exacerbate the risk for subsequent violence and injury (Sigel et al. 2019). This is especially true in cases of intimate partner violence (IPV); when men with a history of IPV perpetration have access to firearms, the risk that they will kill their partner increases by a factor of five (Campbell et al. 2003).

To reduce the risks of firearm violence in the USA, federal law prohibits the purchase and possession of firearms and ammunition for individuals who have been convicted of a felony or a domestic violence misdemeanor (U.S.C.§ 922(g)). Public opinion is largely in favor of these measures (Barry et al. 2018), and evidence supports their effectiveness; denying firearm purchases for individuals with a prohibiting conviction reduces their risk of perpetrating subsequent violent crimes by 25% (Wintemute et al. 2001).

### State-level firearm rights prohibitions

Despite federal prohibitions, most convictions occur in state courts where firearm rights restrictions are implemented based on state law (US DOJ 2011; Love and Schlussel 2021). Only 28 states prohibit people with state-level felony convictions from possessing firearms, and just a subset of those states extend prohibitions to domestic violence misdemeanors (Love and Schlussel 2021; Love 2020). In six states, handgun rights are lost after certain convictions, but long gun rights are not (Love and Schlussel 2021; Love 2020). Given the heterogeneous nature and enforcement of state and federal laws, it is unknown how many people have actually lost their firearm rights from convictions in the USA. Data does suggest that 8% of US adults have been convicted of a felony (Shannon et al. 2017), representing the lower bounds of an estimate for the prevalence of firearm rights suspension.

### Firearm right restoration (FRR)

This commentary focuses on firearm rights restoration (FRR) and the outcomes from such actions. Many states allow prohibited individuals to seek FRR.^1^ States may grant FRR automatically after time is served for certain crimes. In other cases, FRR must be sought through a formal petitioning process, which is the primary focus of this commentary.^2^ These processes are varied. For example, in Illinois, a prohibited person must meet specific requirements based on conviction type, but the decision to grant or deny FRR is left up to a judge’s discretion (ILCS65, 10). On the other hand, Michigan does not allow judicial discretion and instead specifies that FRR should be granted after certain legal criterion are met (MCL 750.224f). Love and Schlussel (2021) attempted to review and summarize FRR state laws but concluded that, “provisions for regaining firearms rights in each U.S. jurisdiction [are] so disparate… [they are] too varied to helpfully compare.”

There are various perspectives regarding how FRR petitioning policies and processes should proceed. Some argue that FRR should be widely available for prohibited persons so that they can more easily re-integrate into society and avoid the collateral consequences of conviction (Love and Schlussel 2021; Whittle 2018). Prohibited individuals may risk re-incarceration if they are found in possession of a firearm even in non-violent circumstances (Shreefter 2017; US Sentencing Commission 2020). On the other hand, many victim-survivors and their advocates argue that FRR should be granted conservatively to minimize threats to public safety (Policy and Center 2000). If a prohibited person were to be granted firearm access, survivors of past violence may be re-victimized or suffer heightened fear for their safety. IPV is characterized by cycles of re-occurring violence, and FRR could enable lethal retaliation by an abuser. As a result, this perspective argues that FRR policies and processes should be designed so that prohibited individuals only become eligible for FRR after it is clear that they no longer poses a threat of harm to others.

## Main text

We reviewed the empirical literature to describe how often FRR petitions are submitted at the state level and whether individuals who were granted FRR engage in subsequent violence. See Additional file 1 for details about the search. After reviewing publications from six online databases, we found no peer-reviewed, empirical research focused on FRR petitioning in the USA.

Through additional search of the grey literature (i.e., not peer-reviewed), we identified three empirical reports on FRR, two had been published over 20 years ago (see Additional file 1: Appendix 1). One report from the US General Accounting Office attempted to collect administrative court data from six states (CA, FL, MA, MI, TX, UT) covering the years 1969-2001, but found the information on FRR was often unavailable or collected irregularly (Ekstrand and Burton 2002). The second report described FRR petitions submitted through a federal mechanism that was discontinued in 1992 (Policy and Center 2000). Currently, most FRR actions are processed at the state-level. The most recent report came from the *New York Times* where journalists accessed data from Washington state and estimated that 3,300 people had been granted FRR between 1995 and 2010, approximately 13% were subsequently were re-arrested (Luo 2011). It was unclear whether these data were comprehensive for the full state and how this crime rate compares to those who did not obtain FRR.

## Conclusion

Research on FRR is needed to establish which legal and procedural approaches to FRR most effectively mitigate the risk of subsequent harms, while equitably reinstating firearms rights to individuals who no longer pose a threat of harm to others. Research is also needed on possible disparities in access to FRR for subgroups of prohibited people, as well as information on who may be disproportionately harmed by FRR in subsequent violence. The legal landscape for FRR is heterogeneous. While there has been some work to synthesize information about state-level FRR laws (Love and Schlussel 2021; Love 2020), these efforts are part of reports focused broadly on restoration of civil and voting rights. Dedicated legal epidemiology work is needed on FRR and firearm prohibitions across states.

Given challenges utilizing administrative data on FRR noted in the grey literature, more systematic documentation of FRR within state court systems may be needed. Researchers could consider building research partnerships with state courts to thoroughly mine existing data systems, or approach policy makers to discuss the feasibility of legally requiring systematic documentation of FRR in a centralized manner. In the absence of systematic data, reviewing hardcopy court records or conducting courtroom observations of FRR hearings within a locality could provide promise. The legal landscape for firearms rights is always evolving, and the research community must provide an evidence base to guide critical decisions related to FRR.

### Supplementary Information

**Additional file 1**. Methods for Systematic Search.

## Data Availability

Not applicable.

## Abbreviations:

DV: Domestic violence
FRR: Firearm rights restoration
IPV: Intimate partner violence
USA: United States

## Footnotes

1 State FRR process work in concert or in contrast with federal prohibitions in heterogeneous ways.

2 Other legal procedures exist which can indirectly restore firearm rights (e.g., expungement, set-aside, or vacated convictions), but these processes are more burdensome on the prohibited person and often require modifying or revisiting the nature of the original conviction.
