# Supplementary material for: Research needs related to firearm rights restoration
Source: Inj Epidemiol. 2024 Jan 5;11:1. doi: 10.1186/s40621-023-00482-1 (PMC10768349; doi:10.1186/s40621-023-00482-1)
Supplement: Supplementary file 1 — Additional file 1. Methods for Systematic Search. [file 40621_2023_482_MOESM1_ESM.docx]

**Methods for Systematic Search**

We collaborated with a team of research librarians at the University of Washington Health Sciences Library to construct a search strategy. We restricted our inclusion criteria to articles that were peer-reviewed, written in English, based in the US, and reported on empirical data about civil FRR petitioning at the state-level among individuals with former criminal convictions. Non-empirical policy analyses, case reviews, or commentaries were excluded.

We searched six online databases using two different search queries, shown in Table 1. We used Covidence (1) to organize results and remove duplicates. Two coders independently screened each title/abstract. Records were retained if they focused on purchase, possession, or personal use of a firearm in the US (i.e., articles on warfare were removed). Articles were subsequently removed if they lacked focus on FRR (e.g., article described firearm purchasing behaviors or opinions in the general US population) or if FRR was connected to an underlying mental health prohibition. If two coders disagreed at this stage, a third coder provided a tie-breaking vote. Finally, non-empirical records were removed.

***Table 1. Systematic search queries***

| Date of search | October, 2022 |
| --- | --- |
| Databases searched | PubMed, Criminal Justice Abstracts, Sociological Abstracts, APA PsycInfo, Academic Search Complete, Web of Science |
| Free-text query 1 | (gun OR firearm OR "second amendment") AND (restor* OR reinstat* OR relief OR regain*) AND (felon* OR ex-felon* OR convict* OR post-convict* OR incarcerated OR misdemean* OR "protective order" OR “restraining order”); |
| Free-text query 2 | (gun OR firearm OR "second amendment") AND (“collateral consequence” OR “collateral sanction” OR (restor* AND right) OR “civil disabilit*” OR (felon* AND right) OR (misdemean* AND right)) |

***Figure 1. Systematic search process***
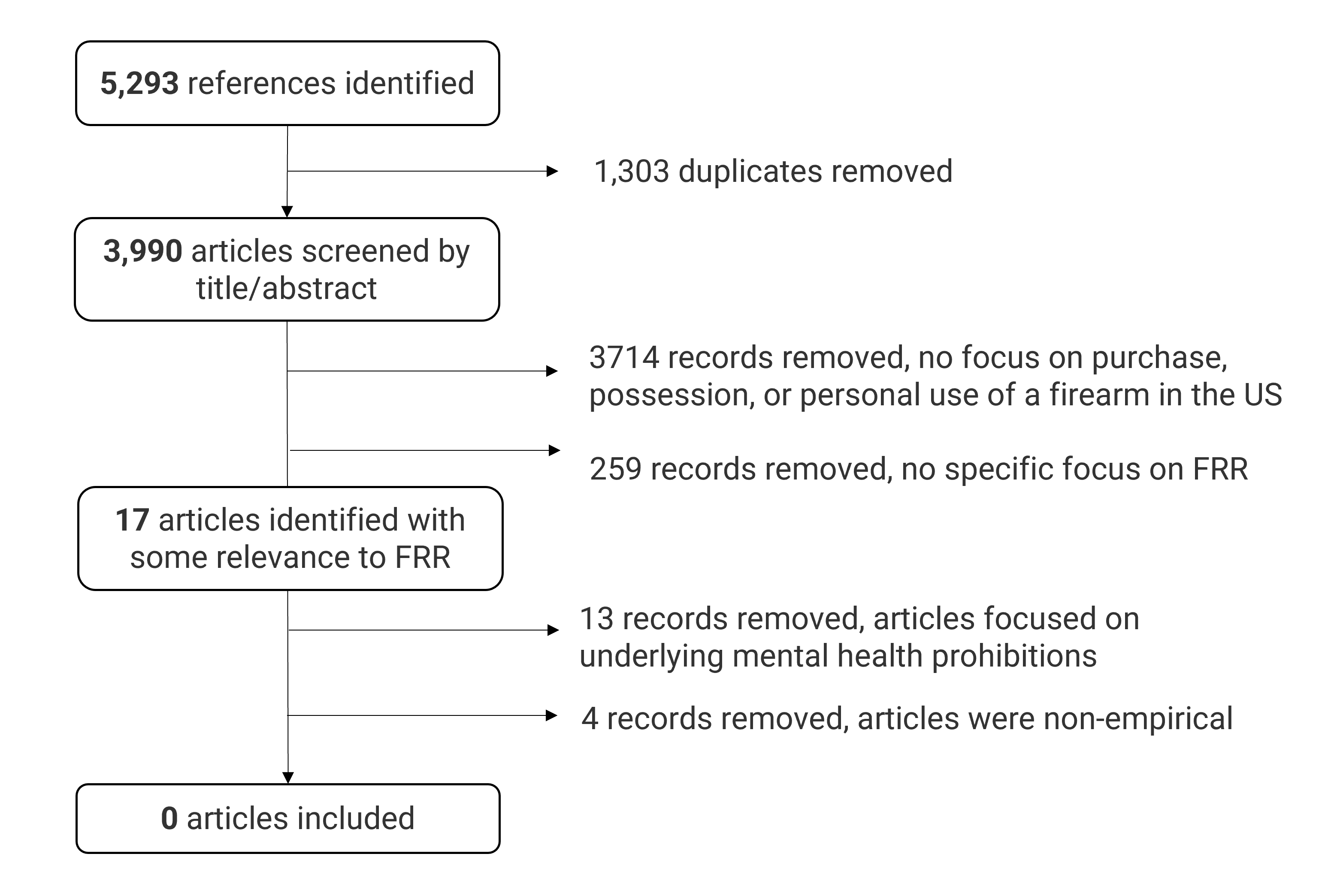


**Results**

The search returned 3,990 unique records, see Figure 1. Seventeen articles discussed FRR. These articles were either related to mental health prohibitions (n=13) and/or were non-empirical (n=4). Across excluded categories, articles primarily discussed laws and their broad implementation (n=11), arguments for/against FRR policies (n=3), or knowledge and attitudes related to FRR (n=3). We found no empirical research on FRR (either federally or at the state-level) that met our inclusion criteria.

Through additional search of the grey literature (i.e., not peer-reviewed, identified through Google searchers and citation mining), we identified three empirical reports on FRR, two of which were over 20 years old as-of the time of writing. These two older reports are described below.

First, the US General Accounting Office collected administrative court data from six states (1969-2001) on granted FRR actions resulting from either direct FRR petitioning, expungements, official pardons, or set asides (2). They found that data was often unavailable or was collected irregularly over time, prohibiting rigorous analyses or generalizability of findings. Still, they reviewed n=538 completed FRR actions and found that only n=3 individuals granted FRR had a subsequent criminal conviction. From state-to-state-, the reporting time period varied dramatically from one to 25 years based on state-level data availability (2). Results suggest that FRR may not be consistently documented in centralized state databases.

Second, the Violence Policy Center reviewed FRR requests that were submitted to the Bureau of Alcohol, Tobacco, Firearms, and Explosives (ATF), 1982-1992 (3). ATF allegedly processed more than 22,000 FRR requests during this 10-year period, of which they estimate about a third were granted. Among those granted FRR petitions from 1985-1992, the Violence Policy Center determined that n=69 individuals were subsequently re-arrested, although it’s unclear how many people were followed and for how long (3). It is important to note that this ATF mechanism for FRR was discontinued in 1992 and is no longer active.

Finally, the article in the New York Times described how many FRR cases were granted in the state of Washington from 1995-2010 (4). Details on the findings are described in the main body of the commentary.

**Sources:**

1. Veritas Health Innovation. Covidence systematic review software. Melbourne, Australia.

2. Ekstrand LE, Burton DR. Opportunities to Close Loopholes in the National Instant Criminal Background Check System. US General Accounting Office; 2002. Contract No.: GAO-02-720.

3. Violence Policy Center. Guns for Felons. 2000.

4. Luo M. Felons finding it easy to regain gun rights. New York Times. 2011.
